# The Link That Binds: The Linker of Hsp70 as a Helm of the Protein’s Function

**DOI:** 10.3390/biom9100543

**Published:** 2019-09-27

**Authors:** Graham Chakafana, Tawanda Zininga, Addmore Shonhai

**Affiliations:** Department of Biochemistry, University of Venda, Private Bags X5050, Thohoyandou 0950, South Africa; grahamchakafana40@gmail.com (G.C.); tzininga@gmail.com (T.Z.)

**Keywords:** heat shock protein 70, linker, allostery, chaperone

## Abstract

The heat shock 70 (Hsp70) family of molecular chaperones plays a central role in maintaining cellular proteostasis. Structurally, Hsp70s are composed of an N-terminal nucleotide binding domain (NBD) which exhibits ATPase activity, and a C-terminal substrate binding domain (SBD). The binding of ATP at the NBD and its subsequent hydrolysis influences the substrate binding affinity of the SBD through allostery. Similarly, peptide binding at the C-terminal SBD stimulates ATP hydrolysis by the N-terminal NBD. Interdomain communication between the NBD and SBD is facilitated by a conserved linker segment. Hsp70s form two main subgroups. Canonical Hsp70 members generally suppress protein aggregation and are also capable of refolding misfolded proteins. Hsp110 members are characterized by an extended lid segment and their function tends to be largely restricted to suppression of protein aggregation. In addition, the latter serve as nucleotide exchange factors (NEFs) of canonical Hsp70s. The linker of the Hsp110 family is less conserved compared to that of the canonical Hsp70 group. In addition, the linker plays a crucial role in defining the functional features of these two groups of Hsp70. Generally, the linker of Hsp70 is quite small and varies in size from seven to thirteen residues. Due to its small size, any sequence variation that Hsp70 exhibits in this motif has a major and unique influence on the function of the protein. Based on sequence data, we observed that canonical Hsp70s possess a linker that is distinct from similar segments present in Hsp110 proteins. In addition, Hsp110 linker motifs from various genera are distinct suggesting that their unique features regulate the flexibility with which the NBD and SBD of these proteins communicate via allostery. The Hsp70 linker modulates various structure-function features of Hsp70 such as its global conformation, affinity for peptide substrate and interaction with co-chaperones. The current review discusses how the unique features of the Hsp70 linker accounts for the functional specialization of this group of molecular chaperones.

## 1. Hsp70 Molecular Chaperones

The heat shock 70 (Hsp70)/*E. coli* Hsp70 (DnaK) family of molecular chaperones are among some of the most conserved proteins [1]. Hsp70s are involved in almost every stage of a protein’s life course, facilitating folding of nascent peptides emerging at the ribosomes, overseeing protein trafficking and translocation across membranes, refolding of misfolded protein and channeling those misfolded beyond repair towards degradation [2,3,4,5,6]. Although Hsp70s are ubiquitous molecules, not all of them are constitutively expressed (heat cognate proteins [Hsc]), but several of them are induced in response to cellular stress. Canonical Hsp70 represented by *E. coli* Hsp70 (DnaK) are capable of suppressing protein misfolding/aggregation as well as refold misfolded proteins [7,8,9]. Due to Hsp70s’ high conservation level across species, functional characteristics of the model *E. coli* DnaK are generally mirrored by other canonical Hsp70s from other species. Hsp70 function is modulated by several co-chaperones whose structure and distribution vary across species, giving rise to functional flexibility [10]. Hence, the co-operation of Hsp70 with co-chaperones propagates the functional valency of these otherwise conserved proteins.

## 2. Hsp70 Structure

**Figure 1:**
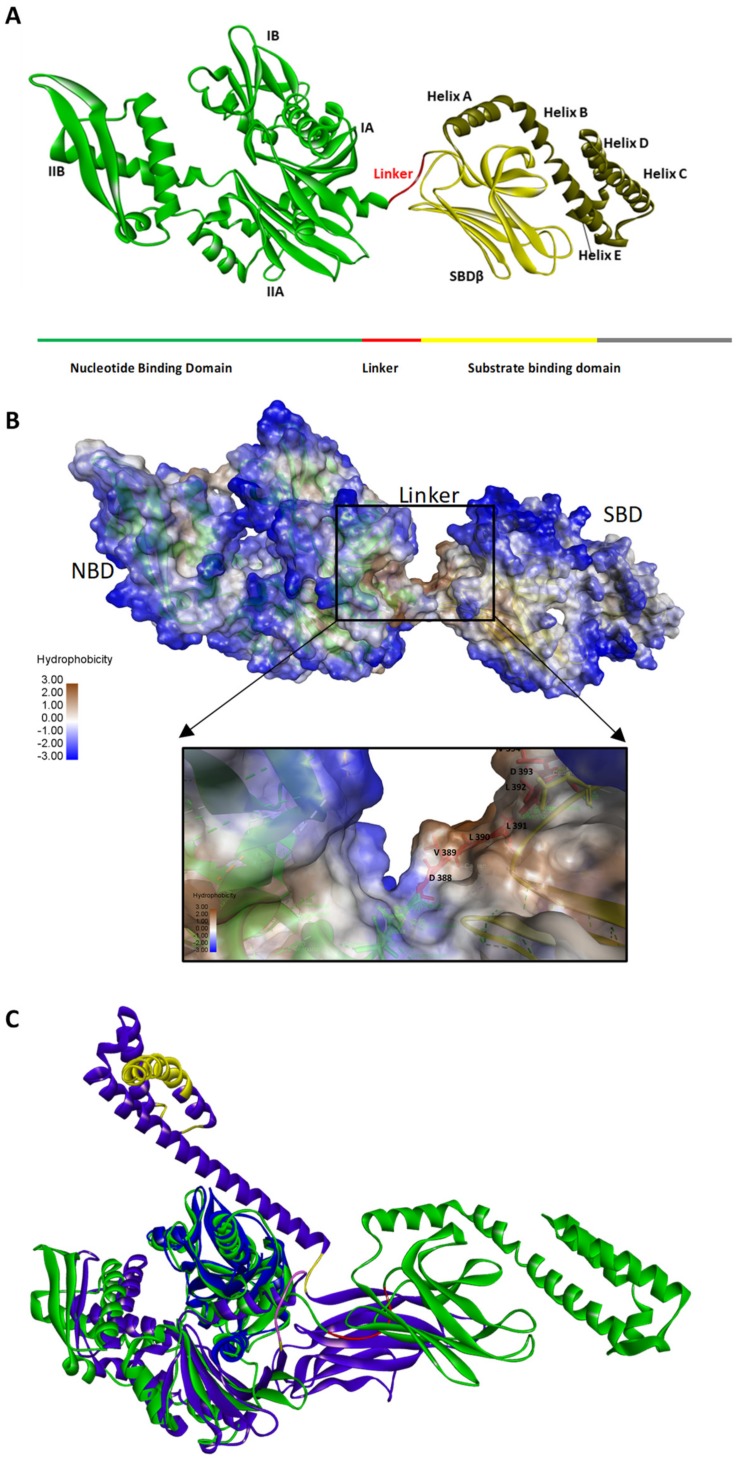
The domain organization of heat shock 70 (Hsp70). Three-dimensional model of *E. coli* Hsp70 (DnaK) showing domain organization (**A**). The N-terminal nucleotide binding domain (NBD) is made up of lobes IA, IIA, IB and IIB (Green). The substrate binding domain (SBD) is made of the SBDβ (yellow) which forms the substrate binding cleft and the SBDα which is characterized by helices A, B, C, D and E (brown). The linker connects the NBD and SBD of Hsp70s (red). The linker of canonical Hsp70s is typically hydrophobic in nature, as confirmed by after hydrophobicity analysis of residues using discovery studio visualizer (https://www.3dsbiovia.com). (**B**) A three-dimensional model of a canonical Hsp70 (green) with the linker highlighted (red). The three-dimensional model of a canonical Hsp70 (P0A648; green) was superimposed against that of Hsp110 (P32589; blue) whose linker (purple) and acidic insertions (yellow) are highlighted (**C**). The modelling was conducted using template C3c7n.1A.pdb [14] on Chimera [15].

The general structure of Hsp70 is made up of a conserved N-terminal nucleotide binding domain (NBD) and a less conserved C-terminal substrate binding domain (SBD) which are connected by a hydrophobic linker (Figure 1). The SBD is composed of two layered twisted β-sandwich (SBDβ) and the SBDα that acts as the lid to SBDβ and promotes the stable binding of substrate (Figure 1A). The NBD of Hsp70s are largely hydrophilic, the SBDβ segment is mostly hydrophobic to facilitate the binding and folding of peptide substrates that typically possess hydrophobic residues (Figure 1B). The Hsp70 superfamily is generally divided into the canonical (DnaK-like) Hsp70s and non-canonical Hsp70s such as the endoplasmic reticulum Hsp70s, Grp78 and Grp170 and the cytosolic Hsp110 members [11,12]. The *E. coli* Hsp70 (DnaK) represents a canonical Hsp70. The linker of DnaK is largely hydrophobic. On the other hand, Hsp110 and Grp170 family members are distinct from canonical Hsp70s in that they possess extended acidic insertions in their SBDs and the C-terminal lid segments [13]. Structurally, canonical Hsp70s and their Hsp110 counterparts share highly conserved NBDs but exhibit sequence variation within the SBDs (Figure 1C). In addition, the linkers of canonical Hsp70s are highly conserved as opposed to those of the Hsp110 subfamily which are more divergent [10,12].

## 3. The General Features of Naturally Occurring Linker Peptides

Most proteins are constituted by globular structures made up of two or more subunits (domains). The domains are connected by ‘linkers’ which are short peptide sequences (2–13 residues in length) [16]. In most proteins, linkers serve as covalent connectors between domains. In addition, they may facilitate interdomain interactions thereby regulating the cooperative function of the domains [17]. The length and amino acid composition of the linker are important determinants of its function. In a study by George and Heringa [18], it was proposed that protein linkers are grouped into small, medium, and large linkers based on average length clusters of 4.5 ± 0.7, 9.1 ± 2.4 and 21.0 ± 7.6 residues, respectively [18]. In addition, longer linkers exhibit higher solvent accessibility, and consistent with this, it was observed that hydrophobicity decreased with increases in linker length, suggesting that longer linkers are more hydrophilic [18]. Altering the length of linkers connecting domains has been shown to affect protein stability, folding rates and interdomain orientations [19,20]. Linkers could also be classified as flexible or rigid based on their amino acid composition [21].

An important feature of linker segments in proteins is to provide flexibility which is crucial for interdomain communication. The flexibility of linkers is based on the rotational freedom of the attached amino acid moieties [22]. Small/polar residues such as threonine and serine typically make flexible linkers due to their small sizes which provide stability due to the capability to form hydrogen bonds with water [21]. Flexible linkers are particularly important when the adjoined domains require a certain degree of movement and/or interaction for efficient allostery [22]. In contrast, rigid linkers (molecular rulers) increase the spatial separation between domains and thus act as tethers [23]. Rigid linkers often contain proline residues where the presence of a cyclic side chain restricts movements and lack of an amide group also prevents hydrogen bonding with joined domains [21].

Based on a database screen of linkers in naturally occurring proteins, threonine (T), serine (S), proline (P), glycine (G), aspartic acid (D), lysine (K), glutamine (Q), asparagine (N) and alanine (A) were suggested to be preferable linker constituents [24]. On the other hand, the study by George and Heringa [18], proposed that proline (P), arginine (R), phenylalanine (F), threonine, glutamic acid (E) and glycine (G) were the most represented. Based on both studies, it was established that polar uncharged or charged residues, were most represented, constituting approximately 50% of naturally encoded amino acids and both studies identified residues P, T and G as the most preferable. As an imino acid, P is a unique residue that imposes restricted flexibility [25]. For this reason, the P residue tends to be well represented in multidomain proteins [26]. On the other hand, the small, polar amino acids, such as T, or S and G tend to provide flexibility to the linker [24]. The study by George and Heringa [18], established that most linkers, on average, exhibited α-helix (38.3%) or coil/bend (37.6%) secondary structures [18]. Based on the study by Argos [24], most linkers (59%) assumed coiled conformations [24]. The study by George and Heringa [18], categorizes linkers into α-helical and non-helical clades [18]. The α-helical linker constitutes a rigid and stable structure that forms rapidly during protein folding [27]. This allows the linker to fold without interfering with the neighboring domains [21]. In addition, the rigidity of α-helical linkers also allows them to space the domains [21]. Non-helical linkers tend to be rich in P residues, which enhances their rigidity, and hence these linkers are effective in reducing interdomain interferences [21]. Overall, most linkers in naturally occurring proteins tend to adopt extended conformations which do not interact with domains present in the proteins [21].

## 4. The Hsp70 Linker Peptides

**Figure 2:**
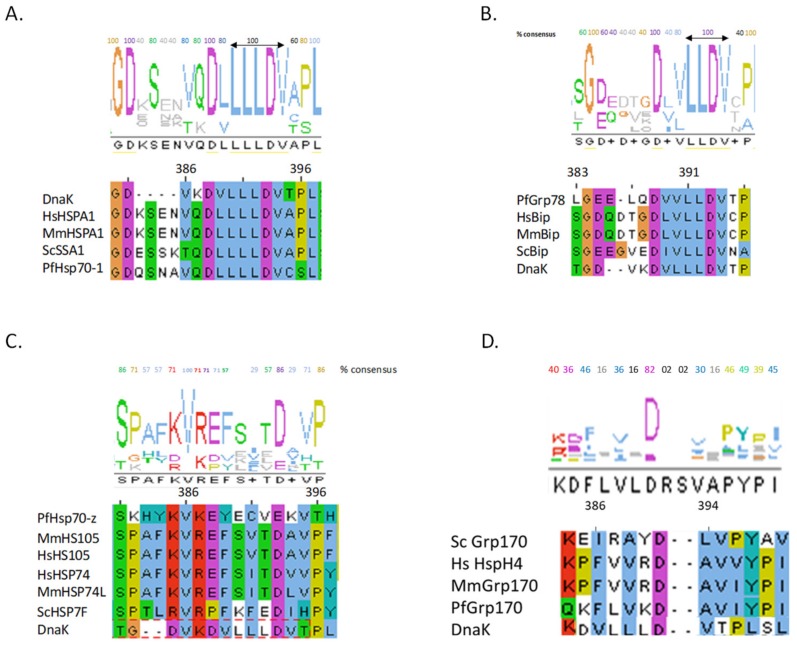
Linker segments of Hsp70s.The data represent linker segments obtained using multiple sequence alignments from at least 500 DnaK homologues and these were sub-grouped based on the conservation level among various Hsp70 clades: (**A**) canonical Hsp70s; (**B**) E.R. Hsp70 homologues; (**C**) Hsp110 homologues and (D) E.R. Grp170 homologues.

The NBD and SBD of canonical Hsp70s are connected by a conserved and highly charged linker peptide, represented by the oligopeptide ^384^GDVKDVLLLLDVT^395^ (residue numbering is based on *E. coli* DnaK) (Figure 2) [28,29]. The residues conserved the most in the linkers of canonical Hsp70s are ^384^GD - - - - D- LLLDV^394^ (Figure 2). On the other hand, the linker of cytosol-localized Hsp70s of eukaryotic origin possesses an insertion of four residues (KSEN/ESSK/QSNA) that are positioned after residue D385 (Figure 2A). The Hsp70 linker is marked by three subsections. The first segment is defined by residues 384–386 (based on *E. coli* DnaK) and is characteristically hydrophilic and of variable length in eukaryotes Table 1, [30]. In addition, this section terminates the NBD of DnaK through residue G384 and has been shown to assume a highly conserved α-helical conformation [30]. The second section is the ^388^DVL^390^, which constitutes a hydrophobic section which assumes an extended β-strand structure [30]. The latter segment confers rigidity to the linker. The third section is comprised of residues ^392^LDV^394^ (Table 1). Upon ATP binding, the ^389^VLL^391^ segment of the linker could be incorporated into subdomain IIA of the NBD forming a small β-sheet [31]. Residues 387–391 of DnaK provide the linker with flexibility [31]. Based on Table 1, although linker residues of Hsp70 members are generally conserved, there is some degree of variation across distinct Hsp70 sub-clusters. This variation within linker residues could impart Hsp70 members with unique functional features given the fact that the linker is a highly structurally adjustable motif which is capable of modulating the global conformation of Hsp70.

The linkers of Hsp70s of eukaryotic origin resident in organelles such as the endoplasmic reticulum (ER) and the mitochondria are to some degree distinct from their cytosolic counterparts based on sequence conservation [32]. In the current review, residues making up the linker of DnaK shall be limited to those positioned at ^385^DVKDVLLLDV^394^. Furthermore, the linker of DnaK was used as a reference for the relative mapping of linker residues of other Hsp70s discussed here using sequence alignment data.

It should be noted that given the short size of the Hsp70 linker segment, any sequence variation within this functionally essential motif has a huge influence on its integrity. Against this background, the residues constituting linkers of canonical Hsp70s generally exhibit high sequence conservation at the N-terminal flank (represented by charged residues ^385^DVKD^388^) and the C-terminal flank (defined by more hydrophobic residues, ^391^LLDV^394^), respectively (Figure 2A). In general, residues on the C-terminal flank of canonical Hsp70 linker segments are hydrophobic (Table 1). On the other hand, residues located on the N-terminus of the linker tend to be charged. This suggests that the Hsp70 linker transmits signals from the N- to the C-terminus and vice versa in distinct fashions. Notably, the linkers of E.R. localized Hsp70s (BiP homologues) exhibit greater sequence conservation on the C-terminal flank than on the N-terminal flank (Figure 2B).

**Figure 3:**
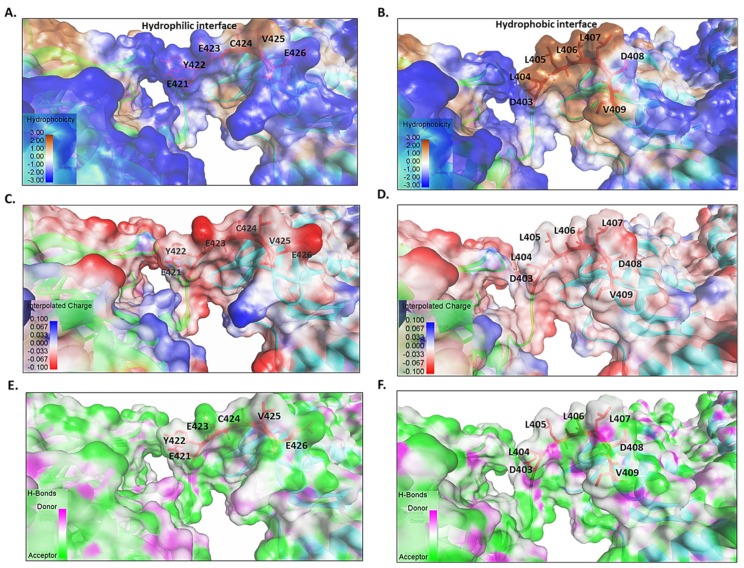
Comparative surface features of linker motifs of PfHsp70-1 (canonical Hsp70) and PfHsp70-z (Hsp110). PfHsp70-z, an Hsp110, possesses a more hydrophilic linker surface interface (**A**) as opposed to the more hydrophobic linker surface of canonical Hsp70, represented by PfHsp70-1 (**B**). The glutamate residues on the surface of PfHsp70-z account for a negatively charged linker surface (**C**) as opposed to that of PfHsp70-1 which is mostly neutral (**D**). The linker of PfHsp70-z (Hsp110) (**E**) possesses more hydrogen binding sites as compared to the linker for the canonical Hsp70 (PfHsp70-1) (**F**).

The linkers of Hsp110 family possess divergent sequences compared to those of other Hsp70 members (Figure 2C). However, the linker residues, 386V and 393D (based on DnaK) are generally conserved across canonical Hsp70 and Hsp110 families (Figure 2C). Multiple sequence analysis of 450 Hsp110 sequences revealed that, in general, Hsp110 linkers cluster into three distinct clades, (EFSVTD, PFKFED and EYECVI) representing Hsp110 members from mammals, yeast and obligate parasites such as *Plasmodium* species, respectively (Figure 2C). It is thus conceivable that the three distinct linker clusters of Hsp110 may define functional specialization of these proteins across various species. This point is best supported by studies based on *Plasmodium falciparum* Hsp70 isoforms. Previously, we observed that the *P. falciparum* cytosol localized Hsp70 (PF3D7_0818900; PfHsp70-1) exhibits ATP-dependent protein aggregation inhibition function, while its Hsp110 counterpart, PfHsp70-z (PF3D7_0708800), exhibits nucleotide-independent function [33,34]. We speculated that this was on account of variation in the flexibility, hydrophobicity, hydrogen bonding and charge variation of the linkers of the two proteins (Figure 3). PfHsp70-1 appears to possess a highly flexible linker as evidenced by the fact that it releases bound substrate instantly upon introduction of ATP in vitro [34]. On the other hand, PfHsp70-z seems to possess a rigid linker and thus is capable of suppressing heat-induced aggregation of model substrate in vitro in a manner that is independent of nucleotide [33,34]. Although both PfHsp70-z and PfHsp70-1 co-localize to the parasite cytosol, PfHsp70-z is more effective at suppressing protein aggregation, hence is thought to act as a buffer against protein misfolding [33] of otherwise aggregation prone proteome of malaria parasites [35]. Linker peptides of canonical Hsp70s and non-canonical Hsp70s possess distinct characteristics that appear to circumscribe the functions of these proteins. For instance, the highly conserved linker of canonical Hsp70s is typically hydrophobic and possesses a neutral charge as compared to the more hydrophilic linkers of Hsp110s (Figure 3).

## 5. Hsp70 Functional Cycle

The function of Hsp70 is regulated by allostery through nucleotide binding at the N-terminal NBD, which in turn influences affinity for substrate. On the other hand, binding of the peptide substrate at the C-terminal SBD is linked to enhanced ATP hydrolysis at the NBD [36,37,38]. This way, the linker of Hsp70 facilitates a two-way signal transmission process. Upon ATP binding, the NBD lobe I rotates relative to lobe II resulting in closure of the nucleotide binding crevice [12]. This facilitates opening of the lower lobes IA and IIA forcing them to make direct contacts with the linker [39]. These events give rise to ATP-induced peptide release from the SBD with concomitant hydrolysis of ATP [28,36,37]. In addition, the rotation of the lobes on the NBD of DnaK displaces two residues (L70 and E171) resident in the catalytic center of the NBD by about 2 Å [37]. ATP binding induces lobe rotations on the NBD forcing the NBD to subsequently dock on the SBD leading to substrate release [6]. Thus, the linker regulates Hsp70 allosteric communication to facilitate substrate release.

**Figure 4:**
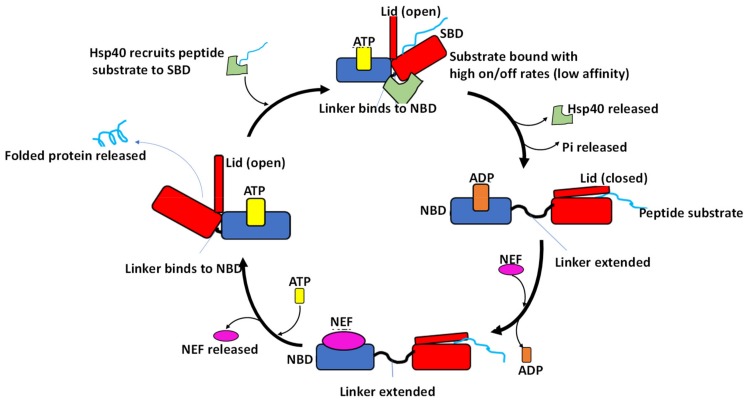
Hsp70 functional cycle. The Hsp40 coupled-protein substrate is delivered to the SBD of the ATP-bound Hsp70. ATP is hydrolyzed and Hsp40 is released from the chaperone complex. Upon Hsp70 binding to ADP, its lid closes to tightly clamp the substrate within the SBD. A nucleotide exchange factor (NEF) facilitates the exchange of ADP for ATP. In the Hsp70-ATP conformation, the lid is open, and the chaperone possesses lower affinity for peptide. This results in the release of the peptide substrate from Hsp70. The fully folded protein is subsequently released. Figure adapted from [44].

Hsp70-mediated protein folding relies on the co-operative action of nucleotides and co-chaperones such as Hsp40 and nucleotide exchange factors (NEFs) (Figure 4). In the ADP/apo state, the NBD and the SBD of Hsp70 are separated from each other by the linker (Figure 4; [40]). In this state, the distance between the nucleotide binding cleft and the substrate binding cleft is reported to be more than 50 Å [10]. For this reason, nucleotide binding by Hsp70 serves to create a platform for allosteric cross talk between the NBD and SBD to regulate substrate binding and release [10,41]. In support of this, data from crystallization of Hsp70 demonstrated that the interdomain interface of Hsp70 is characterized by segments of both the ATPase domain and the SBD, with the helix A of the lid segment (Figure 1) being the most dominant feature [42]. Furthermore, it was suggested that helix A of the lid segment is situated in proximity to the Hsp40 binding site located in the N-terminal ATPase domain of Hsp70 [42]. This would explain why interdomain communication of Hsp70 is a two-way process. Furthermore, residue R171 which is located in the ATPase domain of bovine Hsc70 and is also implicated in Hsp40 binding was further reported to make direct contact with helix A of the lid [42]. This could explain how Hsp40 and the lid may both participate in the allosteric function of Hsp70 [42]. This also further explains how Hsp40 which primarily binds to the N-terminal ATPase domain of Hsp70 is also thought to simultaneously interact with the C-terminus of Hsp70. Since the lid is thought to stabilize substrate binding by Hsp70 [42,43], the role of the linker in modulating the orientation of the lid upon substrate binding would suggest that the lid is indirectly implicated in regulating substrate binding by Hsp70.

In the ATP bound state, Hsp70 exhibits fast on–off rates at the SBD resulting in low affinity for substrate and the ADP bound state is conversely associated with slow on–off rates that generate high affinity for substrate [45,46,47]. The mechanism by which ADP binding at the NBD translates to high affinity state at the SBD is facilitated by the linker whose flexible movement abrogates contacts constituting the NBD–SBDβ interface [48]. Not surprisingly, several residues constituting the NBD–SBDβ interface of DnaK such as R151, R167, D326, V389, D393, K414 and D481 have been implicated in modulating the allosteric function of the protein [49]. Thus, the allosteric function of Hsp70 is facilitated by various residues, which modulate the protein’s conformation with the linker serving as a helm that transmits the signals across the two domains.

It is therefore possible that the interdomain interface of Hsp70 which is characterized by relative positioning of the NBD, SBD and lid regions thus serves as a conduit for the transmission of signals in three distinct facets [50,51]: (i) ATP binding at the NBD leads to signal transmission to the SBD via the linker, leading to NBD and SBD interlocking; (ii) since in the ATP-bound state the lid segment makes contact with the NBD and linker, the lid may directly modulate the NBD and linker to facilitate ATP hydrolysis and (iii) transmission of signal from SBD to NBD via the linker upon peptide substrate binding also modulates the orientation of the NBD. In this sense, the linker serves as an adaptable module connecting the NBD and the SBD [52].

## 6. The Role of the Linker of Hsp70 in Facilitating ATP Binding and Hydrolysis

**Figure 5:**
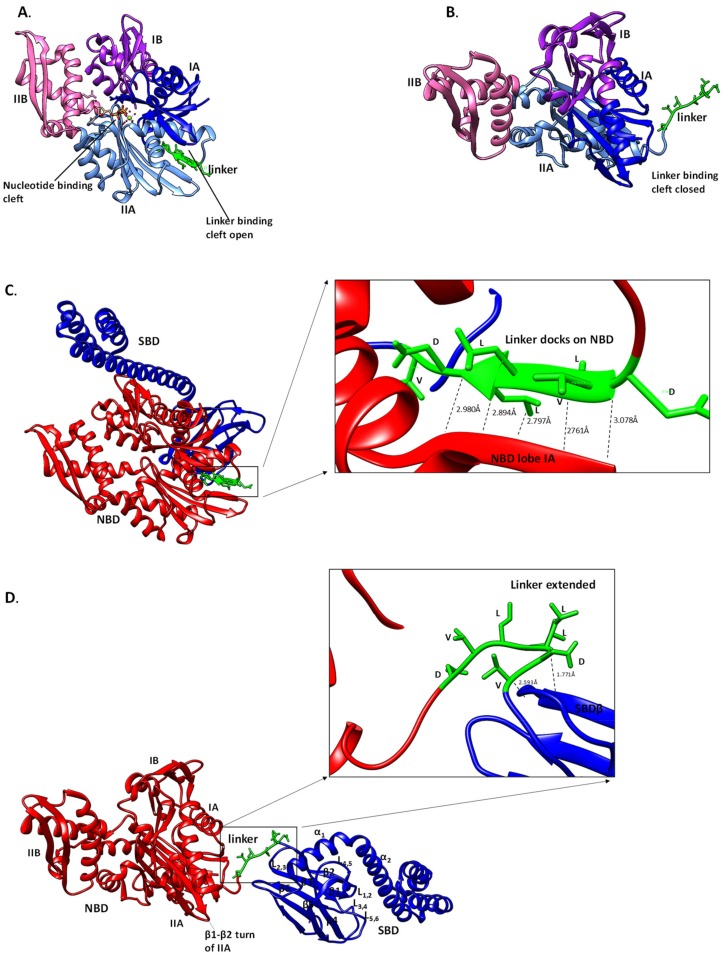
Orientation of linker of Hsp70 in the presence of ATP/ADP. Hsp70 conformation is regulated variably by nucleotides. The conformational changes in the NBD upon ATP binding result in the opening of the linker leading it to docking onto the substrate binding cleft, and ultimately docking onto the NBD (**A**). In the ADP and apo states, the linker binding cleft at the NBD is closed and the linker assumes an extended conformation (**B**). Full length Hsp70 assumes a compact conformation in the ATP-bound state and the linker docks onto the NBD, forming five H bonds (**C**). However, the protein assumes a relaxed conformation in the ADP-bound and apo states, respectively. The linker also assumes an extended conformation forming two H bonds with the SBD (**D**). The templates used for generating the Hsp70 models were: full length Hsp70 in ATP bound form (4po2; [56]), in ADP-bound state (c2khoA; [57]), the NBD in ATP-bound state (c4gniA; [58]), the NBD in ADP bound state (c3iucC; [59]) and NBD in apo state (c4kboA; [60]), respectively.

In the ATP bound state, the lobes IA and IIA of the NBD of Hsp70 move apart to create a solvent accessible crevice onto which the linker fits (Figure 5A,C; [39]). The β-sheet of lobe IIA connects the linker and the nucleotide binding site [31]. The docking of the linker to the edge of lobe IIA’s β-sheet results in a rotation of lobe IIA relative to both lobe IA and the crossing α-helices (Figure 5C; [39]). This, in turn, adjusts the orientation of the β_1_–β_2_ turn in lobe IIA that is responsible for coordinating the γ-phosphate of ATP [39]. Consequently, the ATPase primed form of Hsp70 is sustained by the positioning of the γ-phosphate of ATP [39]. The NBD–SBDβ interface is stabilized by H-bonding [50]. Kityk et al. [51], demonstrated that D481 and K414 both located in the SBD of DnaK make H-bond contacts with lobe IIA of the NBD. This interaction acts as a fastener which facilitates formation of the NBD–SBD interface in the ATP-bound state and subsequently modulates the basal ATPase activity of Hsp70 [46,51].

Substitutions (V389D, L390D and L391D) that were conducted in the linker of DnaK abrogated docking of the linker onto the linker binding cleft in the ATP-bound state [39,52,53]. In addition, substitution of the same linker residues with alanine resulted in loss of ATPase activity [28]. This suggests that the linker is important not only for Hsp70 allosteric function but also modulates the otherwise Hsp70 rate-limiting ATP hydrolysis step [54]. Mechanistically, ATP binding at the NBD nucleotide cleft results in formation of the so-called ‘niche 4L’ motif that is formed when the hydrophilic region of the linker assumes a solvent-exposed loop form [49]). The hydrophobic region of the linker, on the other hand, adopts a β-sheet configuration [30] thus shielding it from the solvent (Figure 5C). In this β-sheet configuration, two leucine residues (L390 and L392) of the DnaK linker face the NBD and participate in hydrophobic packing [30]. Not surprisingly, the highly conserved L390-L393 residues of DnaK are reportedly crucial for ATP hydrolysis [39]. The Hsp70 NBD possessing ^389^VLL^391^ residues from the linker exhibited higher ATPase activity when compared to one that lacked the three residues [39]. In addition, the affinity for ATP exhibited by the NBD possessing the ^389^VLL^391^ residues was comparable to that of the full-length DnaK [39] suggesting an important role of the linker in ATP binding. In another independent study, the linker of both HscA (constitutively expressed Hsp70) and DnaK were shown to autoactivate ATP hydrolysis [55]. This event was shown to depend on the conformational status of Hsp70 which is dictated by the linker residues, ^389^VLL^391^, that assume an extended β-strand (Figure 5) [53,55]. Thus, the orientation of the linker upon ATP binding regulates the NBD to assume a conformation that is most favorable for ATP hydrolysis [54].

In the ADP-bound state, the linker of Hsp70 assumes a relaxed and extended conformation that is undocked from the NBD (Figure 5B,D). In this conformation, the NBD and the SBD function independently of each other [49]. In the ADP state, the linker docking site (on the NBD) becomes more exposed and the relative motion of the NBD and SBD is restricted to a 35° cone [30,56]. It should be noted that, although the linker assumes an extended and relaxed conformation in the ADP-bound state of Hsp70, its flexibility is fairly limited. This rigidity is caused by the formation of the α-helical structure on the NBD-linker interface (Figure 5) [30]. Additionally, in the ADP state, the DnaK linker forms only two H-bonds: L392 with the T417 and V394 with D415 in the SBD (Table 1). Notably, most of the H-bonding observed in the ATP state is largely absent in the ADP bound state of DnaK (Figure 5; Table 2). In the ATP state, the Hsp70 linker makes up to seven hydrogen bonds with the NBD thus facilitating interdomain docking (Figure 5B,D; Table 2). The remarkable capability of the linker to switch between a compact form in the ATP-bound state and a flexible tether in the ADP-bound state underscores its influence on the function of Hsp70.

## 7. The Role of the Hsp70 Linker in Substrate Binding

The SBD of Hsp70 is composed of a two layered twisted β-sandwiches (SBDβ), characterized by a hydrophilic cleft onto which the hydrophobic peptide substrate binds [37,62]. Substrate binding by *E. coli* DnaK occurs via its substrate binding pocket that is constituted by five residues (404M, 427S, 429A, 433Q and 437T) [37,63]. These residues are located in subdomains β1, β3 and β4 of the SBDβ and they bind hydrophobic patches of the substrate that are recognized by Hsp70 [6]. On average, such Hsp70 binding motifs occur every 30–40 residues in virtually all proteins [64]. The bound substrate backbone is then stabilized by α_1_ and α_2_ segments (Figure 5D), and further reinforcement is provided by loop segments, L_1,2_; L_3,4_; L_4,5_ and L_5,6_ [6,37]. Subsequently, the bound substrate is trapped by the ‘closing in’ of SBDα [6,61].

**Figure 6:**
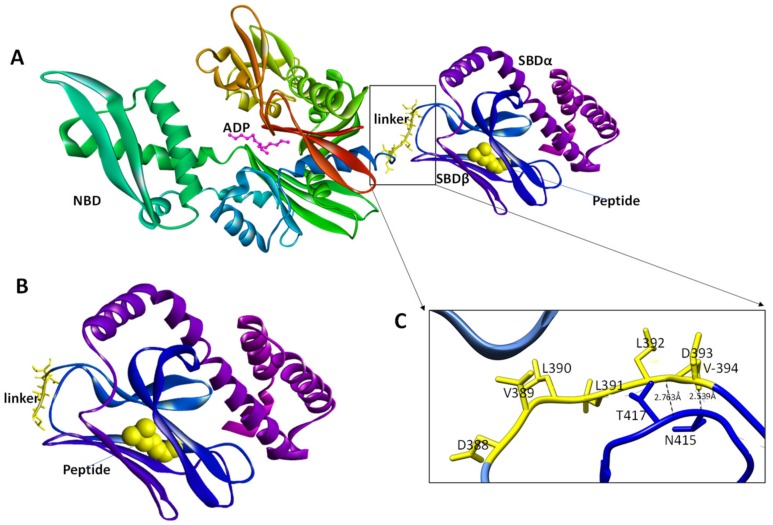
Three-dimensional model of Hsp70 SBD bound to peptide substrate. The middle segment of the linker assumes a coil in ADP bound state (**A**) and the peptide is enclosed into the substrate binding cleft (**B**). The linker forms hydrogen bonds with the SBD in the peptide bound state (**C**). The linker residue V394 with SBD residue N415, while L392 binds T417. The template c2khoA [57] was used for the three-dimensional modelling process.

The association between the SBDβ and SBDα subdomains in the closed conformation is controlled by allostery and substrate binding [37,61]. Thus, cooperation of SBDβ–SBDα segments leads to stabilization of substrate binding. Consequently, these conformational changes result in perturbation of the NBD–SBDβ interface [61]. Thus, upon peptide binding, the linker shifts towards helix A of SBDα, eventually docking into a crevice formed by residues, T398 and P399 of the SBD [65]. This results in the linker assuming a rigid conformation as it ultimately anchors onto the SBD forcing Hsp70 to assume a conformation that is similar to the ATP-bound state [53]. Consequently, this results in reduced conformational freedom of the bond angle (Φ) between linker residues, D393 and V394 (Figure 6; [53]). In the absence of a bound substrate, interaction between the linker and L_2,3_ is destabilized thus enhancing the conformational freedom of the D393–V394 hinge, resulting in collapse of the SBD over the NBD (Figure 6) [40,53]. This mirrors the ATP-bound state which is synonymous with a low affinity for the bound peptide.

Although substrate binding is restricted to the SBD, the resultant intradomain conformational changes on the SBD have far reaching effects on the NBD (Table 3). The linker also interacts with various residues located in the SBD_α_ subdomain [53]. These interactions modulate the linker–NBD interface. This is further enhanced by the direct interaction of the SBD residues, V440 and L484 with D148 on the NBD lobe I [36,51]. The resultant signals eventually reach the catalytic center to initiate ATP hydrolysis. Thus, the linker is important in transmitting signals from the SBD upon substrate binding towards the NBD to facilitate ATP hydrolysis. Indeed, point mutations made on the linker motif have been shown to disrupt ATPase activity induced upon substrate binding [28,66,67]. The overall conformational changes of the SBD and NBD in response to nucleotide binding and/or substrate binding are summarized (Table 3). Generally, ATP binding induces an opening of the linker binding cleft which is accompanied by docking of the linker resulting in reduced flexibility in the NBD and increased flexibility in the SBD (Table 3).

## 8. The Role of the Linker on the Association of Hsp70 with Co-Chaperones

Co-chaperones play an important role in the functional cycle of Hsp70s (Figure 4). The most prominent co-chaperones implicated in regulating the Hsp70 ATPase activity are Hsp40s (called DnaJ proteins in prokaryotes). Hsp40s recruit substrates for Hsp70, and simultaneously stimulate the ATPase activity of the latter [68]. Direct interaction of Hsp40 and Hsp70 is through a highly conserved Hsp40 segment, the J-domain [55]. The J-domain docks onto the Hsp70 linker binding cleft between the NBD lobes IA and IIA [67]. The binding of the J-domain disrupts the direct association of the NBD and the SBD. This allows the SBD to flip freely allowing it to capture the substrate recruited by Hsp40 [69]. Furthermore, the linker is reported to stabilize the Hsp70–Hsp40 functional association [36,67,70]). Kumar et al. [36] observed that mutations L390D, L391D and L392D as well as L390A, 391A and L392A mutations in DnaK resulted in a 10-fold reduction in DnaJ binding affinity. It is thus plausible that, in the absence of the linker, Hsp70 and Hsp40 may fail to form a stable and functional complex. Thus, the linker serves as a co-chaperone docking interface and further transmits signals from substrate binding on the SBD to facilitate Hsp40-mediated ATP hydrolysis.

It has further been reported that DnaK possessing linker mutations, L391S and L392G, failed to co-operate with DnaJ and the *E. coli* nucleotide exchange factor, GrpE to refold denatured luciferase in vitro [71]. This shows that the linker is not only important in the co-operation of Hsp70s with Hsp40 as it also facilitates engagement of the chaperone with other co-chaperones such as nucleotide exchange factors. Kumar and co-workers [36] reported that substitutions, L390[A/D], L391[A/D] and L392[A/D] that were introduced in the DnaK linker abrogated the chaperone’s function in cellulo. In a similar assay, Vogel et al. [28] demonstrated that DnaK with linker mutations 393[A/D] and 393[A/D] introduced failed to complement a DnaK minus *E. coli* strain. This suggests that the function of the linker is essential for the cyto-protective role of DnaK.

Hsp90 and Hsp70 cooperate to fold some proteins such as kinases, transcription factors and steroid hormone receptors that are implicated in cellular development [72]. In addition, Hsp70 and Hsp90 occur in functional networks with several co-chaperones. The functional interaction between Hsp70 and Hsp90 is modulated by Hsp70–Hsp90 organizing protein (Hop/Sti1; [73]), which functions as an adaptor protein linking Hsp90 and Hsp70. Both Hsp90 and Hsp70 each possess a C-terminal EEVD motif that facilitates their interaction with Hop. Hop in turn possesses three tetratrico-peptide repeat domains (TPR1, TPR2A and TPR2B; [73]) that are important for interaction with both chaperones. Initially, Hsp70 bound to a partially folded substrate interacts with Hop via the TPR1 domain, and this allows the TPR2A domain of Hop to access Hsp90. The concomitant conformational changes associated with this leads to the migration of Hsp70 from TPR1 domain to the TPR2B domain of Hop [74]. The transition of Hsp70 to the TPR2B domain is linked to substrate transfer to Hsp90 [74]. Notably, the reorientation of Hsp70 to allow substrate handover to Hsp90 is nucleotide dependent and facilitated by allostery [74,75]. Thus, the linker of Hsp70 is central to the conformational changes that the chaperone undergoes during its interaction with Hsp90. In support of this, Hsp70 inhibitors targeting its N-terminal ATPase domain are known to abrogate its association with Hop via the C-terminal EEVD motif [76,77]. Since Hsp70 and Hsp90 are known to interact with multiple co-factors and client substrates, their association relies on the orientation of the Hsp70 linker. This makes the Hsp70 linker a nexus for the Hsp70-Hsp90 protein folding pathway.

## 9. The Role of the Linker of Hsp70 in Regulating Oligomerization

It is known that Hsp70 self-associates to form dimers or higher order oligomers [78,79,80,81,82]. Oligomerization of Hsp70 is thought to regulate the cellular availability of the functional monomeric form of the protein [82]. In addition, oligomerization also regulates the interaction of Hsp70 with other chaperones such Hsp90 [83,84] and co-chaperones such as Hsp40 [85]. The exact mechanism by which Hsp70 self-associates is a subject of debate. There are several conflicting ideas on the Hsp70 residues that facilitate its oligomerization. In addition, the role of nucleotides on Hsp70 oligomerization remains to be fully elucidated. However, some studies reported DnaK to form oligomers in the ADP bound state and apo state [78,79,86]. The DnaK dimers were reportedly disrupted in the presence of ATP [86]. The oligomerization of bovine Hsc70 is thought to occur through a region in the SBD defined by residues 385–540 [80]. Residues (554–646) located in the C-terminal loop of rat Hsc70 [87] and human Hsp70 [88] are also implicated in the self-association of these proteins. Interestingly, another study reported dimer formation to occur through the NBD–SBD interface (residues 382-561) of human Hsp70 [89], suggesting a possible role of the linker in this event. In support of this, Aprille et al. [82], reported that dimer formation by Hsp70 occurs through interaction of the C-terminal loop of a molecule of Hsp70 and the linker segment of its partner monomer to facilitate self-association. Some studies reported that ATP promotes formation of Hsp70 dimers in antiparallel fashion [37,40,84,85,90]. The reported differences in dimer contact points may suggest that the Hsp70 self-association is species specific. However, notably, the linker, and hence the allosteric function of Hsp70 both seem to play a part in its self-association.

## 10. The Role of the Linker of Hsp70 in Regulating Its Stability

The Hsp70 linker has been implicated in the structural stability of the protein. An NBD of DnaK lacking the linker has been reported to be less stable and less active than an NBD coupled to linker residues, ^390^LLL^393^ [29,36]. This indicates that the linker confers stability and functional integrity to the ATPase domain of the protein. Mitochondrial Hsp70s are reported to exhibit a propensity to aggregate and their linker serves as a sensor that modulates response to environmental changes [91]. Mutations on the linker of mitochondrial Hsp70 reduced the stability of the protein [91]. In another study, the linker and the C-terminal helix of the SBD were both shown to influence stability of the protein [92].

In addition, the linker also acts as a ‘potentiometer’ which helps Hsp70s to sense pH changes in the cellular environment. Using electrospray ionization mass spectroscopy, Swain [29], showed that the NBD attached to a linker segment was more responsive to pH shifts in comparison to the NBD lacking the linker. It is proposed that at neutral pH, the Hsp70/DnaK linker assumes a compact status forcing the NBD to assume a closed state, thus activating the NBD to hydrolyze ATP, while higher pH levels abrogate this effect [29]. Taken together, this confirms that the linker is important in stabilizing Hsp70 and effects this through its ability to modulate the global conformation of the protein.

## 11. Targeting the Linker in Drug Discovery

**Figure 7:**
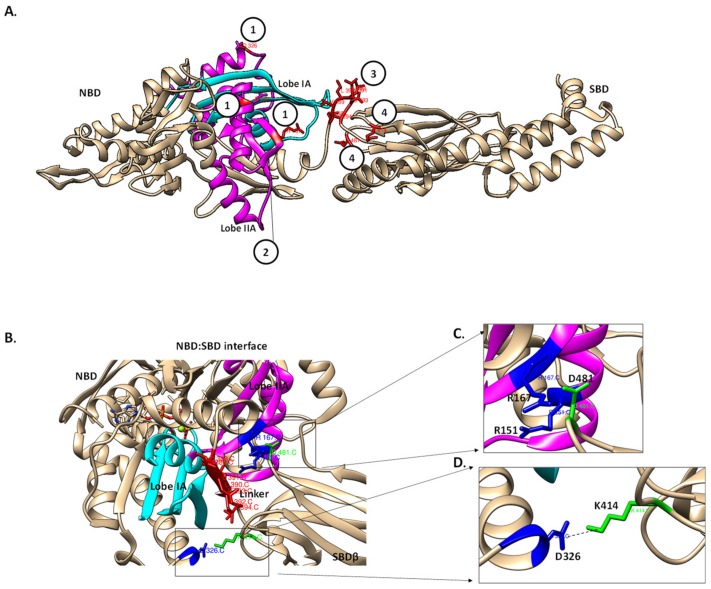
The potential Hsp70 allosteric hotspots for drug targeting. The model of Hsp70 potential drug target sites that are modulated by the linker. (**A**) Hsp70 major allosteric hotspots located are shown in red. (1) NBD residues involved in the formation of the NBD–SBD interface (R151, R167 and D326 based on DnaK numbering); (2) the linker binding cleft between lobe IA (magenta) and IIA (cyan) form the hydrophobic linker binding cleft which is crucial for linker docking in the ATP state; (3) linker residues such as V389 and D393, hence, are also crucial for the NBD–SBD interface formation and (4) SBD residues, K414 and D481 (based on DnaK) that are crucial in the formation of the NBD–SBD interface [51]. (**B**) The NBD–SBD interface is shown with the allosteric hotspots in the NBD (blue) and SBD (green), respectively. The linker docked onto the linker binding cleft facilitates the formation of the NBD–SBD interface. NBD residues R167, R151 (blue) and the SBD (green) residue D481 form a part of NBD–SBD [51] (**C**), while NBD residues, D326 and K414 interact through hydrogen bonding thus stabilizing the interface (**D**) [51].

Hsp70s have been proposed as potential therapeutic targets [93,94,95,96]. Some prospective anti-cancer drugs targeting Hsp70 have entered the clinical phase [97,98]. However, the main limitation of these prospective pharmacological agents has been their reported toxicity to normal cells [97]. In spite of this, a promising approach would be to identify compounds that abrogate the functional network of Hsp70. To this end, the NBD–SBD interface could present an ideal target site for small molecule inhibitors of Hsp70 (Figure 7). Such inhibitors may abrogate the allosteric function of the protein. Hsp70 also presents a prospective target against infectious agents such as malaria parasites [98,99]. However, selectively targeting this otherwise highly conserved molecule across species possesses a major challenge. Since the linker occupies the NBD–SBD interface, it is part of a structural platform that brings together the highly conserved NBD and the more structurally diverse SBD of Hsp70. For this reason, the linker and the NBD–SBD interface of Hsp70 could constitute a structurally unique site which could be selectively targeted by small molecule inhibitors that are specific for a given Hsp70 (Figure 7B).

The flexibility of the Hsp70 linker is a defining character of the protein’s function. For example, canonical Hsp70s possess a highly flexible linker which robustly transmits signals emanating from the N-terminal ATPase domain (NBD) to the remotely positioned C-terminal SBD and vice-versa. Not surprisingly, some inhibitors of Hsp70 targeting the ATPase domain have been shown to abrogate interaction of the chaperone with Hop, a co-chaperone that primarily binds to the C-terminal EEVD motif of Hsp70 [76,77,100]. This suggests that the linker is capable of transmitting signals that perturb the global conformation of the Hsp70 in response to inhibition of the protein by small molecule inhibitors irrespective of their specific binding site. In this way, the linker acts as a chink in the armor of the protein. The fact that linkers of Hsp70s that fall under the Hsp110 clade appear less flexible, compared to the conserved linker present in canonical Hsp70s, suggests that inhibitors of Hsp70 that affect its global conformation may be more effective against canonical Hsp70s than they are against Hsp110 members. In this way, the linker of Hsp70 is not only a crucial determinant of the protein’s normal function but may present a unique structure that could be selectively targeted by small molecule inhibitors to abrogate function of specific members of this otherwise conserved family of proteins. For this reason, it is essential to establish Hsp70 inhibitors which would most effectively modulate the conformation of the protein to abrogate its myriad of functions such as ATPase activity, chaperone function and association with co-chaperones/other chaperones.

## 12. Conclusions

The structural conformation of Hsp70 is important for its functions such as ATP hydrolysis, substrate binding, stress response, structural integrity and oligomerization. Interdomain communication is integral to the chaperone function of Hsp70. In the current review, we discussed the role of the linker in Hsp70 proteins. We further established that linker segments in non-canonical Hsp110 members of the Hsp70 family are less conserved and are delineated into at least three distinct clades based on the sequence conservation. It is important to experimentally validate how the unique features of these linkers define the functional features of the proteins. What is clear is that the linker of Hsp70 represents a structural helm that regulates the protein’s global conformation and function. The linker may thus be amenable to Hsp70 drug targeting in several disease models. There are two main possible ways to target the Hsp70 linker. One approach would be to identify small molecule inhibitors of Hsp70 that target the ATPase–linker–SBD interface. Alternatively, an indirect approach would be to identify small molecule inhibitors that primarily target either the ATPase or SBD, leading to linker reorientation thereby abrogating association of Hsp70 with its co-chaperones and/or substrates.

## Figures and Tables

**Table 1:** Properties of Amino Acid Residues of Linkers of Canonical Hsp70s.

	Cytosolic Hsp70s *	E.R. Localized Grp78	Cytosolic Hsp110s	Grp170s
First Section	**G384** (non-polar, hydrophobic)	**G** (non-polar, hydrophobic)	**P** (non-polar, hydrophobic)	**K** (polar, positively charged, hydrophilic)
	**D385** (polar, negatively charged, hydrophilic)	**D** (polar, negatively charged, hydrophilic)	**K** (polar, positively charged, hydrophilic)	**S** (polar, uncharged, hydrophilic)
	**V386** (non-polar, hydrophobic)	**G** (non-polar, hydrophobic)	**V** (non-polar, hydrophobic)	**E** (polar, negatively charged, hydrophilic)
Eukaryotic Insertion	**K** (polar, positively charged, hydrophilic)	-	-	-
	**S** (polar, uncharged, hydrophilic)	**E** (polar, negatively charged, hydrophilic)	-	-
	**E** (polar, negatively charged, hydrophilic)	**D** (polar, negatively charged, hydrophilic)	**A** (non-polar, hydrophobic)	**E** (polar, negatively charged, hydrophilic)
	**N** (polar, uncharged, hydrophilic)	**T** (polar, uncharged, hydrophilic)	**F** (non-polar, hydrophobic)	**V** (non-polar, hydrophobic)
Hinge	**Q387** (polar, uncharged, hydrophilic)	**G** (non-polar, hydrophobic)	**K** (polar, positively charged, hydrophilic)	**K** (polar, positively charged, hydrophilic)
Second Section	**D388** (polar, negatively charged, hydrophilic)	**D** (polar, negatively charged, hydrophilic)	**E** (polar, negatively charged, hydrophilic)	**D** (polar, negatively charged, hydrophilic)
	**L389** (non-polar, hydrophobic)	**L** (non-polar, hydrophobic)	**F** (non-polar, hydrophobic)	**F** (non-polar, hydrophobic)
	**L390** (non-polar, hydrophobic)	**V** (non-polar, hydrophobic)	**S** (polar, uncharged, hydrophilic)	**L** (non-polar, hydrophobic)
Hinge	**L391** (non-polar, hydrophobic)	**L** (non-polar, hydrophobic)	**V** (non-polar, hydrophobic)	**V** (non-polar, hydrophobic)
Third Section	**L392** (non-polar, hydrophobic)	**L** (non-polar, hydrophobic)	**T** (polar, uncharged, hydrophilic)	**L** (non-polar, hydrophobic)
	**D393** (polar, negatively charged, hydrophilic)	**D** (polar, negatively charged, hydrophilic)	**D** (polar, negatively charged, hydrophilic)	**D** (polar, negatively charged, hydrophilic)
	**V394** (non-polar, hydrophobic)	**V** (non-polar, hydrophobic)	**G** (non-polar, hydrophobic)	**V** (non-polar, hydrophobic)

* Numbering based on *E. coli* DnaK (P0A648). The residues used in this analysis represent the most frequently occurring residues identified from multiple sequence analysis of approximately 450 sequences using JalView (https://www.jalview.org).

**Table 2:** Linker residues involved in Hydrogen bond formation upon ATP/ADP binding.

Nucleotide	Linker Residues	Contact Residues [Domain]	H Bond Length/Å
ATP	D388 (O)	K214 (N) [NBD]	3.078
	L390 (N)	K214 (O) [NBD]	2.761
	L390 (O)	F216 (N) [NBD]	2.797
	L392 (N)	F216 (O) [NBD]	2.894
	L392 (O)	V218 (N) [NBD]	2.980
	D393 (O)	I418 (N) [SBD]	2.913
	D393 (O.D)	V394(N) [linker]	2.794
ADP	L392 (O)	T417 (H) [SBD]	1.771
	V394 (N)	D415 (O) [SBD]	2.593

The predicted bond length values were obtained using protein 3-dimensional models generated using Chimera version 1.1. The templates used for generating the Hsp70 models were as follows: full length Hsp70 in ATP bound form (4po2; [61]), or in ADP-bound state (c2khoA [57]), respectively.

**Table 3:** Summary of allosteric activity of Hsp70s.

Bound Nucleotide/Substrate		Subdomain Conformation					Overall Subdomain Dynamics	
NBD	SBD	Nucleotide binding cleft	Linker binding cleft	Linker	SBD	Lid	NBD	SBD
ATP	-	closed	open	docked	docked	released	rigid	flexible^(a)^
ADP	-	open	closed	mobile	undocked	docked	flexible	rigid^(b)^
-	peptide	open	closed	mobile	undocked	docked	flexible	rigid^(c)^
ADP	peptide	open	closed	mobile	undocked	docked	flexible	flexible^(d)^
ATP	peptide	closed	open	docked	docked	released	rigid	flexible^(e)^
-	-	open	closed	mobile	undocked	released	flexible	flexible^(f)^

Legends: NBD Nucleotide binding domain, SBD-Substrate binding domain, NB cleft-Nucleotide binding cleft, IA-IIA- NBD lobes IA and IIA. References in superscript a- [53]; b- [54], c- [52], d- [30], e- [49].
